# Advances in deciphering the mechanisms of salt tolerance in Maize

**DOI:** 10.1080/15592324.2025.2479513

**Published:** 2025-03-18

**Authors:** Xiaofei He, Junke Zhu, Xuehua Gong, Dongqing Zhang, Yuan Li, Xiansheng Zhang, Xiangyu Zhao, Chao Zhou

**Affiliations:** a State Key Laboratory of Crop Biology, College of Life Sciences, Shandong Agricultural University, Taian, Shandong, China; b School of Agricultural Engineering & Food Science, Shandong University of Technology, Zibo, Shandong, China; c College of Life Sciences, Qilu Normal University, Jinan, Shandong, China; d Hebei Province Carbon-Based Heavy Metal Soil Pollution Remediation Technology Innovation Center, Tangshan, Hebei, China

**Keywords:** Maize, salt stress, reactive oxygen species (ROS), plant hormones, ions

## Abstract

Maize (*Zea mays* L.) is a vital crop worldwide, serving as a cornerstone for food security, livestock feed, and biofuel production. However, its cultivation is increasingly jeopardized by environmental challenges, notably soil salinization, which severely constrains growth, yield, and quality. To combat salinity stress, maize employs an array of adaptive mechanisms, including enhanced antioxidant enzyme activity and modulated plant hormone levels, which work synergistically to maintain reactive oxygen species (ROS) balance and ion homeostasis. This review explores the intricate interactions among ROS, antioxidant systems, plant hormones, and ion regulation in maize under salt stress, providing a comprehensive understanding of the physiological and molecular basis of its tolerance. By elucidating these mechanisms, this study contributes to the development of salt-tolerant maize varieties and informs innovative strategies to sustain agricultural productivity under adverse environmental conditions, offering significant theoretical insights into plant stress biology and practical solutions for achieving sustainable agriculture amidst global climate challenges.

## 1. Introduction

Maize, recognized as one of the most significant and widely cultivated crops, is believed to have been domesticated from its wild ancestor, teosinte, in Mexico around 7,000 years ago.^1^ Since then, it has evolved into a highly versatile crop that plays a pivotal role in global agriculture.^2^ Maize is utilized in a variety of forms, serving as a staple food source, an essential component of animal feed, and a raw material for numerous industrial products. These include ethanol for biofuel, starch for food and non-food applications, and snack foods such as popcorn, making it indispensable for both food security and industrial applications.^3,4^ In recent years, the rapid growth of the global population has significantly heightened the demand for food, feed, and biofuel, placing immense pressure on agricultural systems to improve crop yield and quality. Meeting this demand has become a critical global challenge, particularly in the face of environmental constraints. Among these, soil salinization has emerged as a formidable barrier to agricultural productivity.^5–7^ This environmental stress affects plant growth, reduces crop yields, and poses a severe threat to food security. The severity of this issue is further compounded by factors such as environmental degradation, unsustainable irrigation practices, and the ongoing effects of climate change. Given the extent of the problem, developing crops, particularly maize, that can withstand the challenges posed by saline soils is essential for sustaining food production in affected regions.^8,9^

Salt stress disrupts plant metabolic processes and impairs growth by causing osmotic stress, ionic toxicity, and oxidative damage.^10^ These adverse effects lead to cellular dysfunction, nutrient imbalances, and inhibition of photosynthesis and other critical metabolic pathways.^11^ In response, plants have evolved a suite of physiological and biochemical strategies to cope with saline environments.^12^ These include enhancing the activity of antioxidant enzyme systems, such as superoxide dismutase (SOD), catalase (CAT), and peroxidase (POD), to counteract the damage caused by reactive oxygen species (ROS).^13,14^ Additionally, plants modulate the levels of key phytohormones, including abscisic acid (ABA), ethylene, and cytokinins (CKs), to mediate stress responses and adapt to salinity.^15,16^

Selective ion uptake and compartmentalization also play a pivotal role in maintaining ionic homeostasis, ensuring that essential ions are retained while excess sodium (Na^+^) and chloride (Cl^−^) ions are sequestered in vacuoles or excluded.^17,18^ Furthermore, the synthesis of osmolytes, such as proline, glycine betaine, and soluble sugars, helps plants adjust their osmotic potential to restore cellular water balance. These mechanisms collectively mitigate the primary and secondary damage induced by salt stress and support plant survival under salinized conditions.

This review delves into the complex interplay between ROS, antioxidant enzymes, phytohormones, ion transport systems, and osmotic regulation in maize, highlighting the strategies to perceive and transduce salt stress signals. Particular attention is given to the molecular mechanisms underpinning ion transport and signaling pathways, which are critical for maize adaptation to saline environments. Understanding these regulatory networks is essential for improving crop productivity under stress conditions and holds substantial theoretical and practical significance for developing salt-tolerant maize varieties through genetic and agronomic interventions.

## 2. Role of antioxidant enzymes and ROS in maize salt tolerance

ROS are highly reactive molecules that contain oxygen and play a crucial role in cellular metabolism across various living organisms. These molecules include free radicals, such as superoxide anion radicals (O^2−^) and hydroxyl radicals (OH), as well as non-radical oxidants like hydrogen peroxide (H_2_O_2_) and singlet oxygen.^13^ Salt stress, in particular, induces excessive ROS production, which can significantly hinder plant growth and development.^14,19^ This is one of the major limiting factors for crop productivity, especially in regions affected by high soil salinity.^20^ Under salt stress, the overproduction of ROS leads to oxidative damage, which affects various cellular components such as lipids, proteins, nucleic acids, and membranes.^21^ This oxidative damage compromises cell integrity and function, impairing essential physiological processes like photosynthesis, respiration, and nutrient transport. The imbalance in ROS levels can disrupt cellular ionic homeostasis by interfering with the functioning of ion channels and pumps, leading to cellular dysfunction. Additionally, excessive ROS can cause lipid peroxidation in cellular membranes, damaging membrane structures and causing leaks of essential cellular contents,^22,23^ ROS can also oxidize proteins and nucleic acids, leading to enzyme inactivation, protein aggregation, and DNA mutations, all of which further contribute to cellular stress and plant growth inhibition.^24^

**Figure 1.**
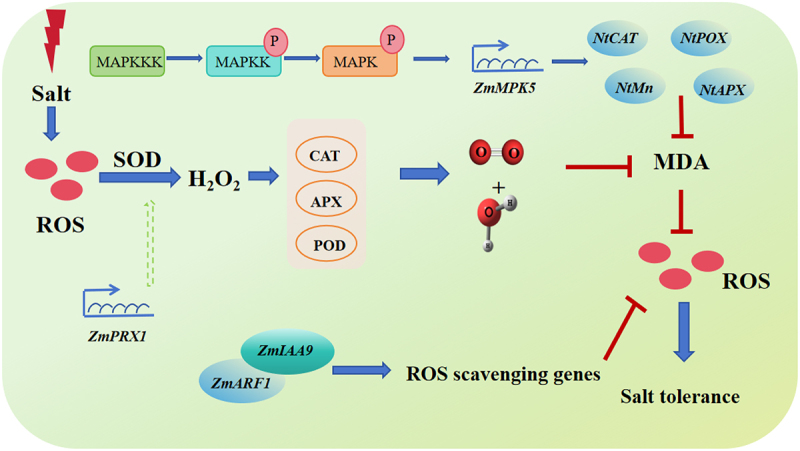
The process of ROS production and balance under salt stress. Reactive oxygen species (ROS) are produced as a result of salt stress, and their accumulation can lead to oxidative damage in plant cells. SOD, CAT, ascorbate peroxidase (APX), and POD are key antioxidant enzymes that play crucial roles in scavenging ROS and maintaining cellular redox balance. SOD catalyzes the dismutation of superoxide anion radicals into hydrogen peroxide, which is then detoxified by CAT and APX.

To mitigate the damaging effects of ROS, plants have developed a variety of antioxidant defense mechanisms to scavenge and neutralize excess ROS.^25^ These mechanisms involve both enzymatic and non-enzymatic components. For example, antioxidant enzymes such as SOD, CAT, and POD play key roles in converting harmful ROS into less reactive molecules.^26,27^ In maize, a well-studied mechanism involves the Aux/IAA gene family. The gene ZmIAA9, in particular, has been shown to interact with the downstream transcription factor ZmARF1 to regulate the expression of ROS scavenging genes.^28^ This interaction helps to enhance the plant’s capacity to clear excess ROS, improving its ability to cope with salt stress and reducing the associated oxidative damage. Recent studies have also demonstrated that exogenous application of ROS scavengers, such as antioxidants or enzymes, can significantly reduce the negative impacts of water stress and salt stress on maize. For instance, applying ROS scavengers can improve the photosynthetic efficiency of maize leaves by decreasing the degradation rate of chlorophyll. This, in turn, enhances the net photosynthetic rate, which is critical for maintaining energy production and growth under stressful conditions. The application of these scavengers has been shown to improve the overall health of maize plants, leading to enhanced tolerance to both salt and drought stress.^29^ These findings underscore the importance of ROS management in improving crop resilience to environmental stresses, and they offer potential strategies for enhancing the productivity of crops like maize in challenging growing conditions (Figure 1).

Under stress conditions, maize crops accumulate significant amounts of ROS, which triggers an increase in antioxidant enzyme activity and the accumulation of osmotic regulators.^30,31^ These processes help mitigate oxidative stress by clearing excess ROS. The primary antioxidant enzymes include SOD, CAT, POD, glutathione peroxidase (GPX), glutathione reductase (GR), and APX.^32,33^ SOD serves as the first line of defense in the plant antioxidant system, converting accumulated superoxide molecules into oxygen and hydrogen peroxide (H_2_O_2_). CAT, APX, and POD then further break down H_2_O_2_ into water and oxygen.^34^ Together, these enzymes also remove malondialdehyde (MDA), a byproduct of lipid peroxidation, thus protecting cellular membranes from oxidative damage.^35^ Antioxidants, such as glutathione (GSH), ascorbic acid (ASA), mannitol, flavonoids, and anthocyanins, play essential roles in maintaining the balance of ROS within the cell.^36^ These compounds are distributed throughout the cell and help regulate oxidative stress.^37^ The combined action of antioxidant enzymes and antioxidants effectively reduces the damage caused by salt and alkali stress. Research has shown that SOD levels in maize increase with higher salt concentrations, particularly during the three-leaf, flowering, and grain-filling stages. POD activity also rises during the flowering and grain-filling stages in response to increased salt stress. CAT activity shows the most significant changes during the grain-filling stage, initially increasing before decreasing once a threshold is reached.^38^ The PRX gene plays a crucial role in the removal of excess free radicals in plants and is recognized as one of the key enzymes in the plant’s defense response to stress. In maize, *ZmPRX1* induces the expression of POD, SOD, and CAT, contributing to the clearance of excess ROS, promoting the accumulation of osmotic regulators, and reducing cell membrane damage, thereby enhancing the plant’s resistance to stress.^39^ The increased activity of antioxidant enzymes and glutathione reductase (GR) plays an important role in enhancing plant salt tolerance.^40^ Overexpression of certain antioxidant enzyme genes has also demonstrated the critical role of these enzymes in responding to salt stress. Research has reported a significant relationship between antioxidant capacity and salt tolerance in cotton. Meneguzzo and colleagues attributed the better salt tolerance of one wheat variety compared to another to the induction of ascorbate synthesis.^41^ Phospholipid hydroperoxide glutathione peroxidase (PHGPX) has been found in salt-tolerant citrus cells. Plants may utilize other mechanisms to help balance ROS levels and manage the energy consumed within the plant.^42^ These processes could involve events such as leaf curling, and the reorganization of photosynthetic apparatus.^43^

Reactive oxygen species (ROS) are essential in maize’s response to various environmental stresses, especially drought. One of the plant’s primary responses to drought is stomatal closure, which helps conserve water by reducing transpiration. This process is controlled by turgor pressure within the guard cells, which surround the stomata. Three key signaling molecules-abscisic acid (ABA), ROS, and calcium ions (Ca^2+^)-play critical roles in regulating stomatal closure under drought stress.^44^ When the plant detects water scarcity, ABA is quickly synthesized in the roots and transported to the guard cells. In the guard cells, ABA binds to the PYR receptor protein, which inhibits the activity of PP2C phosphatase.^45^ This inhibition lifts the suppression of SnRK, activating it. Activated SnRK then triggers the production and movement of extracellular ROS into the guard cells, which further contribute to the closure process.^46,47^

The HPCA1 receptor kinase on the guard cell surface detects extracellular hydrogen peroxide (H₂O₂), a type of ROS. This detection activates calcium channels in the guard cell membrane, resulting in an increase in intracellular Ca^2+^ levels. The rise in Ca^2 +^ concentration then signals the guard cells to close the stomata, thereby helping the plant conserve water during drought. Additionally, the WRKY transcription factor ZmWRKY106 is significantly upregulated in maize under heat and drought conditions. ZmWRKY106 modulates the ABA signaling pathway, regulating the expression of stress-related genes such as RD29A, HSP90, and DREB2A. This transcription factor plays a key role in enhancing maize’s tolerance to both drought and heat stress. Overexpressing ZmWRKY106 leads to an increase in the activities of antioxidant enzymes such as superoxide dismutase (SOD), peroxidase (POD), and catalase (CAT). These enzymes help mitigate oxidative damage by reducing ROS levels, suggesting that ZmWRKY106 contributes to the plant’s ability to manage oxidative stress during adverse conditions. In summary, ZmWRKY106 plays a crucial role in maize’s response to drought and heat by regulating antioxidant defenses and modulating key stress-related pathways.^48^

In response to salinity stress, transcription factors serve as key intermediaries, linking external signals with the regulation of genes that help the plant resist stress. These factors receive signals from upstream pathways and bind to specific regions of DNA, called cis-regulatory elements, to control the expression of downstream genes that are involved in the stress response. Among the many transcription factor families in plants, the NAC (NAM, ATAF1/2, and CUC2) family is one of the largest and most diverse. NAC transcription factors are involved in a wide range of physiological processes, including the synthesis of important compounds like proline and starch, hormone regulation, and responses to abiotic stresses such as drought, cold, and salinity.^49^

Several NAC genes in maize have been found to enhance drought tolerance when expressed in transgenic Arabidopsis plants. Notable examples include ZmSNAC1, ZmNAC33, and ZmNAC55, which help improve the plant’s ability to cope with water scarcity.^50^ More recently, the maize gene ZmNAC074 has been identified as being upregulated under heat stress. ZmNAC074 enhances the plant’s heat tolerance by activating genes involved in scavenging reactive oxygen species (ROS) and other stress response pathways.^51^ Another important maize NAC gene, ZmNAC89, plays a significant role in salt tolerance. When ZmNAC89 is overexpressed in transgenic maize, the plants exhibit improved resistance to salinity. This is achieved through the modulation of redox and abscisic acid (ABA) signaling pathways, which help maintain cellular homeostasis and protect the plant from salt-induced damage. Together, these NAC transcription factors are essential for maize’s ability to withstand various abiotic stresses, including salinity, by regulating key stress-related pathways.^52^

The mitogen-activated protein kinase (MAPK) cascade is a key signaling pathway that involves a series of three interconnected protein kinases: MAPKKK (MAP kinase kinase kinase), MAPKK (MAP kinase kinase), and MAPK (MAP kinase). The process begins when receptors on the cell surface perceive external signals, such as stress cues, and relay these signals through intermediate molecules. This activation triggers the first kinase in the cascade, MAPKKK, which then phosphorylates MAPKK. MAPKK, a dual-specificity kinase, further activates MAPK by adding phosphate groups to conserved threonine (T) and tyrosine (Y) residues.^53^ This three-tier kinase system is highly conserved across eukaryotes and plays a fundamental role in regulating various cellular processes, including plant growth and responses to environmental stresses such as drought, heat, and salinity.^54^

Once activated, the MAPK cascade ultimately phosphorylates specific effector proteins, which then trigger cellular responses to help the plant adapt to stress. In maize, a salt-induced MAPK called ZmSIMK1 has been identified as a crucial player in enhancing salt tolerance. Overexpression of ZmSIMK1 in maize results in the upregulation of stress-responsive genes, such as RD29A and P5CS1, which are involved in stress adaptation, and enhances the plant’s ability to tolerate salt stress.^55^

Similarly, another MAPK gene, ZmMPK5, is induced in maize under salt stress. When ZmMPK5 is overexpressed in transgenic tobacco plants, these plants show reduced accumulation of reactive oxygen species (ROS) compared to wild-type plants under salt stress.^56^ This is accompanied by an increased expression of ROS-related genes, such as NtCAT (catalase), NtPOX (peroxidase), and NtAPX (ascorbate peroxidase), suggesting that ZmMPK5 plays a significant role in mitigating oxidative stress by regulating antioxidant defense mechanisms. Furthermore, overexpression of ZmMPK5 leads to the upregulation of various stress-responsive genes, including those involved in abscisic acid (ABA) signaling (e.g., NtNCED1), polyamine synthesis (e.g., NtADC1 and NtSAMDC), proline metabolism (e.g., NtP5CS), and stress defense proteins (e.g., NtERD10C and NtERD10D).^57^ These changes collectively contribute to enhanced salt tolerance.

Additionally, ABA signaling, which is crucial in plant responses to drought and salt stress, has been shown to increase in response to these stress conditions. This heightened ABA signaling further activates MAPK-related genes, reinforcing the plant’s stress tolerance. In summary, the MAPK cascade, through the regulation of key genes involved in oxidative stress response, ABA signaling, and other adaptive mechanisms, plays a vital role in enhancing the ability of plants like maize to withstand abiotic stresses such as salinity.

## 3. The functions of plant hormones in modulating salt tolerance mechanisms in maize

When plants are continuously exposed to various external environmental factors such as drought, heat, cold, high salinity, or attacks by pathogens/pests, their growth and reproductive capacity are severely affected.^58^ Therefore, plants must possess the ability to sense and quickly respond to these external conditions to adapt to the ever-changing environment.^59^ Plant hormones are compounds synthesized by plants that regulate various aspects of plant growth and development.^60^ These small molecules, derived from secondary metabolism, play a crucial role in helping plants adapt to environmental stimuli.^61,62^ which form an interconnected communication network to help plants adapt to changing environments.^63^

Under high salinity stress, the level of abscisic acid (ABA) in plants increases.^64^ ABA is a 15-carbon weak acid that was first discovered in the 1960s. It acts as a growth inhibitor and has been shown to affect many aspects of plant growth and development, such as embryo maturation, cell division and elongation, seed dormancy and germination, flower induction, and responses to environmental stress.^65^ Previous studies have found that salt stress induces ABA accumulation in maize lateral root primordia, leading to disrupted polar distribution of auxin.^66^ which affects root growth. Further research has identified oxidative stress induced by salt stress as the main cause of ABA accumulation.^67^ ABA is an important hormone in regulating plant growth and development, as well as in response to abiotic stresses such as drought and high salinity. During the salt stress response, ABA synthesis is rapidly induced, leading to a swift increase in ABA levels.^68^ High levels of ABA activate kinase cascades and improve stress recognition and defense responses.^69^ Salt stress limits water absorption, resulting in cell dehydration and changes in cell expansion, creating osmotic stress. Under high salinity conditions, the increase in endogenous ABA levels leads to stomatal closure to regulate water balance and osmotic homeostasis.^70^ Therefore, osmotic regulation is an important function of ABA-mediated plant salt stress response.^71^ Previous studies identified *ZmPRR37* gene, that affects maize flowering and salt tolerance. The promoter of this gene contains multiple ABA-responsive elements. Research has shown that the *ZmPRR37* gene knockout mutant is sensitive to salt stress, while overexpression of *ZmPRR37* enhances maize salt stress resistance. Exogenous application of ABA can reduce the sensitivity of the *ZmPRR37* gene knockout mutant to salt stress. ZmPRR37 can directly bind to the promoter of the ABA-responsive gene *ZmDhn1*, inhibiting its transcription and thereby enhancing maize’s tolerance to salt stress.^72^

In the absence of abscisic acid (ABA), PP2C phosphatases act to inhibit the activity of SnRK kinases, which are critical for stress responses. However, under conditions of abiotic stress, such as salt stress, ABA receptor proteins – namely pyrabactin resistance 1 (PYR1), PYR1-like (PYL), and regulatory components of ABA receptors (RCAR) – bind to the accumulated ABA. This interaction causes the inhibition of PP2C phosphatase activity, thereby relieving the suppression of SnRK kinases. As a result, a cascade of kinases, including Raf-like protein kinases and SnRK2s, is activated. This cascade plays a crucial role in the early stages of osmotic regulation in response to salt stress.^73^

Furthermore, ABA signaling triggered by salt stress directly targets ABA response elements (ABREs) in the promoter regions of specific genes. This interaction leads to the upregulation of various stress-related genes. Once activated, SnRK kinases also stimulate the AREB/ABF transcription factors, which regulate the expression of downstream genes involved in stress tolerance. These processes collectively enhance the plant’s ability to cope with salt stress by improving its osmotic regulation and activating key stress-responsive pathways.

Jasmonates (JAs) are lipid-derived endogenous hormones and are key regulators in plant developmental processes and various defense responses.^74,75^ Studies have shown that JA synthesis-deficient mutants have larger stomatal diameters in their leaves under salt stress compared to the maize wild-type B73, indicating that JA promotes the movement of guard cells under salt stress to reduce water loss. Chlorophyll content measurements and leaf senescence phenotyping revealed that, compared to B73, JA synthesis-deficient mutants exhibit delayed leaf senescence under salt stress, suggesting that JA also plays an important role in salt-induced programmed cell death and leaf senescence.^76^ Exogenous application of JA can alleviate the harmful effects of NaCl stress by enhancing antioxidant enzyme activity and the ability to scavenge free radicals.^77^ After salt treatment, JA synthesis-deficient mutants produce less H_2_O_2_ in their leaves compared to B73, but higher levels in their roots. SOD, CAT, and APX activities also show similar patterns, with the enzyme activities in the leaves of the JA synthesis-deficient mutants being lower than B73, while the root enzyme activities are higher. Regarding osmotic regulation, JA synthesis-deficient mutants accumulate less proline in their leaves under 100 and 300 mm NaCl treatment, but produce more proline in their roots under all salt concentrations compared to B73.^78^ This result suggests that JA synthesis-deficient mutants experience greater oxidative stress and osmotic stress in their roots under salt stress, while their leaves are under less stress than the wild-type. Additionally, JA may positively regulate the biosynthesis of ABA in the leaves under salt stress.^79^ In support of this, a study reported the role of JA in improving salt stress induced by Na_2_CO_3_ JA-treated maize seedlings significantly reduced the toxic effects of Na_2_CO_3_ by decreasing Na^+^ absorption, ROS accumulation, and MDA levels.^80^

Coronatine-insensitive 1 (COI1) is a critical receptor in the jasmonic acid (JA) signaling pathway, characterized by leucine-rich repeats (LRR) and an F-box domain.^81^ Initially discovered in *Arabidopsis thaliana*, COI1 proteins are also present in maize, where they are classified into two subgroups: COI1 and COI2. Under salt stress, maize produces signaling molecules such as jasmonic acid, which binds to the COI1 receptor. This binding activates COI1, enabling it to interact with and bind to JAZ proteins, forming the SCFCOI1-JAZ complex.

As a component of the SCF E3 ubiquitin ligase complex, COI1 facilitates the ubiquitination of JAZ proteins. This modification marks the JAZ proteins for degradation by the 26S proteasome. The degradation of JAZ proteins releases their repression of jasmonic acid-responsive genes, allowing the MYC2 transcription factor to become active. MYC2 then regulates the expression of various downstream genes involved in the plant’s stress response.^76^ This signaling cascade plays a pivotal role in maize’s ability to respond to salt stress by regulating the expression of genes that help the plant adapt to changing environmental conditions.^82^

Gibberellin (GA) is a key endogenous plant growth regulator that plays a crucial role in regulating various physiological processes, including seed germination, stem elongation, and flowering.^83^ Under stress conditions, such as salt stress, the application of gibberellin (GA3) has been shown to alleviate the detrimental effects of salt-induced damage in plants.^84,85^ In maize, the application of GA3 has been shown to significantly promote plant growth under salt stress conditions. One of the primary ways in which GA3 mitigates salt stress is by reducing the levels of harmful ROS, such as hydrogen peroxide (H_2_O_2_) and superoxide anions (O^2−^), which accumulate under salt stress. Additionally, GA3 treatment lowers the concentration of sodium ions (Na^+^) within plant tissues, which helps restore ion balance and reduces the toxic effects of salt accumulation. GA3 has been shown to upregulate the activity of enzymes such as catalase (CAT), superoxide dismutase (SOD), and peroxidase (POD), which are involved in the detoxification of ROS, and has been observed to increase potassium (K^+^) concentrations in maize plants.^86^ Potassium is essential for maintaining osmotic balance and cellular function, particularly under salt stress. The higher K^+^ levels promote better water retention and cellular turgor, contributing to improved salt tolerance. In previous studies, GA3 treatment has been found to reduce the levels of MDA and H_2_O_2_, and trigger the activity of glutathione-S-transferase (GST), an enzyme involved in the detoxification of various harmful compounds, including ROS and toxic metabolites.

The GA-deficient mutant ga1–3 has long been recognized for its remarkable tolerance to salt stress.^87^ When compared to wild-type plants, seedlings that lack the gibberellin (GA) signaling repressors GAI, RGA, RGL1, and RGL2 exhibit less growth inhibition under salt stress conditions.^88^ This suggests that the absence of these repressors helps improve the plant’s ability to withstand the negative effects of salinity. Furthermore, the overexpression of the GA catabolic gene *CYP71D8L* has been shown to enhance salt tolerance by modulating the balance of GA levels within the plant. By regulating GA levels, CYP71D8L helps optimize the plant’s response to stress, contributing to better growth and survival under salt stress.^89^ These findings highlight the importance of GA signaling in managing salt stress and suggest potential avenues for improving salt tolerance in crops through genetic manipulation of GA-related pathways.

Brassinosteroids (BRs) are a class of polyhydroxy steroid plant hormones that play a critical role in regulating various physiological processes, including cell elongation, division, and differentiation, as well as the regulation of stress responses, metabolism, and reproductive development.^90,91^ The molecular mechanisms by which BRs exert their effects primarily involve a signaling cascade that is mediated through specific receptor proteins, most notably the BRI1 (BRI1-EMS suppressor 1), and further amplified by transcription factors such as BES1 (BRI1-EMS suppressor 1) and BZR1 (brassinazole-resistant 1), which are critical components of the BR signaling pathway.^92,93^ In maize, the brassinosteroid signaling pathway is also a significant regulator of growth under both normal and stress conditions.^94^ A particular transcription factor, *ZmBES1*, has been identified as a critical player in maize’s response to external environmental factors. ZmBES1 is localized in the nucleus, where it functions as a key regulator of gene expression in response to salt stress. Heterologous expression of *ZmBES1* in maize has revealed several significant effects. One of the most notable findings is that the expression of *ZmBES1* reduces the plant’s sensitivity to ABA, which in turn promotes more robust growth under stress conditions. This reduction in ABA sensitivity helps the plant adapt to abiotic stresses by mitigating the negative effects of stress-induced growth inhibition. Additionally, the expression of *ZmBES1* promotes the growth of both the stem and root system, which are crucial for the plant’s ability to anchor itself and absorb nutrients and water, particularly under stress conditions. These enhanced growth patterns provide maize plants with a better capacity to withstand environmental challenges, improving their overall performance. Moreover, overexpression of *ZmBES1* in maize has been shown to confer increased salt tolerance. This effect is partially due to a reduction in malondialdehyde (MDA) content, a marker of oxidative stress and cell membrane damage. By reducing MDA levels, the plant minimizes oxidative damage, thereby improving cellular integrity and promoting better survival under high-salinity conditions. Salt stress in plants often leads to the accumulation of ROS, which can damage cellular structures like membranes and proteins. By modulating the response to oxidative stress, *ZmBES1* helps maize plants better manage ROS levels, improving their ability to cope with salt-induced damage. In addition to *ZmBES1*, the maize brassinosteroid signaling kinase gene, *ZmBSK1*, has also been implicated in the response to salt stress.^95^ ZmBSK1 acts as a crucial mediator in the brassinosteroid signaling pathway, and its expression is upregulated in maize leaves, roots, and stems in response to NaCl treatment. This upregulation indicates that ZmBSK1 plays a critical role in modulating the salt stress response, likely through the activation of downstream stress-response pathways.^96,97^

Brassinosteroids (BRs) and abscisic acid (ABA) work synergistically to regulate plant salt tolerance by coordinating various signaling pathways.^98^ BRs are recognized by the receptor BRI1, which then activates the coreceptor BAK1 and the kinase BSU1. This activation inhibits the BIN2 kinase and, in turn, promotes the activity of the BZR1/BES1 transcription factors, which regulate the expression of genes involved in plant growth and stress responses. Additionally, BIN2 plays a role in activating ABA signaling by phosphorylating SnRK2.2 and SnRK2.3, key kinases involved in ABA responses.^99^

Conversely, the phosphatases ABI1 and ABI2 act as negative regulators in ABA signaling. These phosphatases dephosphorylate BIN2, which reduces its activity and, consequently, enhances BR signaling. This interplay between BR and ABA pathways ensures a balanced response to salt stress, where BRs promote growth and stress adaptation, while ABA signaling helps the plant manage water loss and osmotic balance. Together, these hormonal interactions contribute to the plant’s ability to cope with the adverse effects of salt stress by regulating both growth processes and stress response mechanisms.

**Figure 2.**
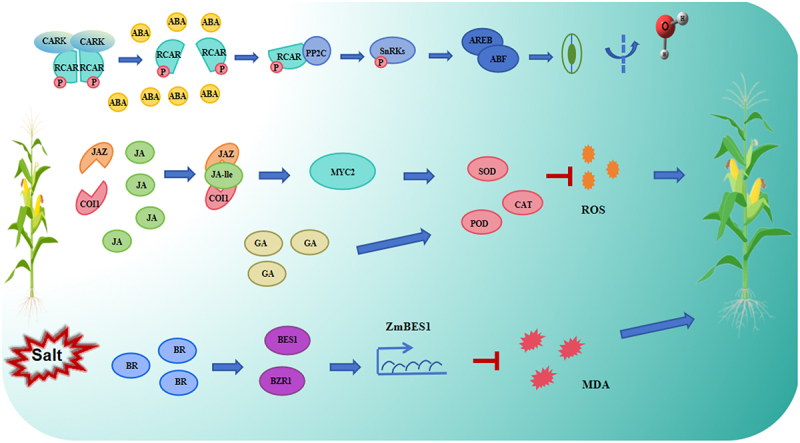
The regulatory network of plant hormones in regulating salt tolerance in maize. The blue arrows indicate positive effects, while the red lines represent inhibitory effects. BR, GA, JA, and ABA are key plant hormones involved in mediating the response to salt stress. These hormones function in an intricate regulatory network to enhance or inhibit various cellular and physiological processes. ABA plays a critical role in stress signaling, promoting stomatal closure and inducing stress-related gene expression. JA is involved in the modulation of stress-responsive genes and the activation of defense mechanisms. GA interacts with stress signaling pathways to adjust growth under salt conditions. BRs help modulate growth and stress responses, balancing the plant development under salt stress.

Plant hormones such as ABA, JA, GA, and BRs play important roles in mediating salt stress signaling and balancing the response to salt stress (Figure 2). Some plant hormones act as positive regulators in plant salt tolerance, while others have negative effects. Despite the well-documented roles of these hormones, our understanding of how they specifically interact and regulate salt stress mechanisms in maize remains limited. For instance, how do plant hormones coordinate the regulation of ROS homeostasis under salt stress? Additionally, How do plant hormones regulate ion transport? These questions highlight the gaps in our current knowledge, underscoring the need for further research into the precise roles of plant hormones in regulating maize’s response to salt stress.

## 4. Ion transport in the Regulation of Maize Salt Tolerance

Plants need to maintain the dynamic balance required for intracellular physiological metabolism through selective absorption, efflux, and ion compartmentalization. The content and dynamic balance of Na^+^, K^+^, and Cl^−^ ions are crucial factors in preventing salt stress.^100,101^

Na^+^ is one of the most abundant soluble cations in saline soils.^102,103^ Under salt stress, high concentrations of sodium ions in the soil disrupt the dynamic balance of intracellular ions, causing a range of detrimental effects on plants, such as damage to cell membrane structure, abnormal cellular metabolism, and ion toxicity.^104,105^ Therefore, maintaining the internal balance of Na^+^ is critical for plants to resist salt stress.^5,106^ Mechanisms to reduce cytoplasmic Na^+^ include limiting Na^+^ uptake, enhancing Na^+^ efflux, and compartmentalizing Na^+^ into vacuoles.^107,108^ HKT1 is considered a key regulator of plant salt tolerance under salt stress.^109^ HKT1 may improve salt tolerance by reducing Na^+^ accumulation in stem tissues, thus protecting leaves from Na^+^ toxicity.^110,111^ *ZmNC3*, encoding the plasma membrane-localized HKT family ion transporter ZmHKT1;2, has inward Na^+^ transport activity and enhances salt tolerance in maize by reducing Na^+^ transport from xylem to aerial parts, thereby lowering Na^+^ accumulation in aboveground tissues.^112^ Mutations in HKT1 suppress the salt-sensitive phenotypes of *SOS2* and *SOS3* mutants, indicating that HKT1 and the SOS pathway jointly regulate Na^+^/K^+^ homeostasis in plant cells.^113–115^ Genome-wide association studies (GWASs) and quantitative trait locus (QTL) analyses have identified genetic variations related to salt tolerance in plants, enabling the cloning of numerous HKT loci in different plant species. This highlights the critical role of HKT proteins in the evolution of plant salt tolerance and their importance in crop breeding.

**Figure 3.**
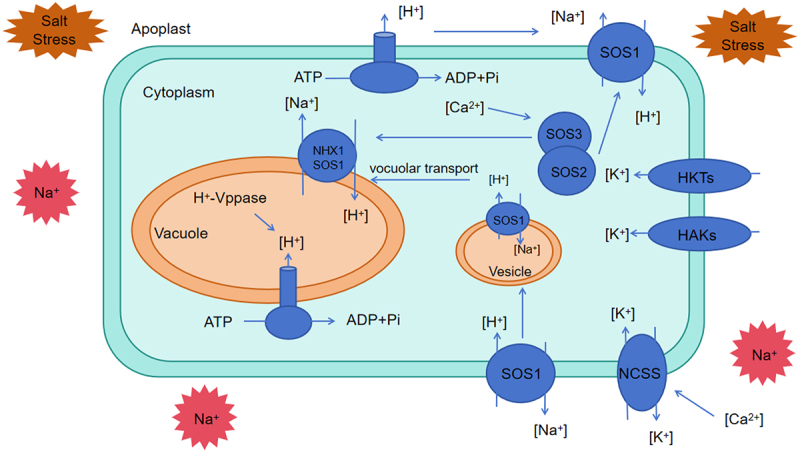
Maintaining ionic homeostasis in the plant cells under salt stress. The Na^+^ ions are primarily excreted through the SOS1 transporter in the plasma membrane and the NHX1 transporter in the vacuolar membrane. The SOS1 activity is further enhanced by the activation of the SOS2/SOS3 signaling complex. In addition to the active transport of Na^+^, the H^+^ gradient across membranes, generated by H^+^-ATPase and H^+^-V-ATPase pumps, provides the necessary driving force for the transport process. Na^+^ ions can be transported out of the cell through vesicles to further maintain cellular homeostasis. In the process, K^+^ also play a significant role in counterbalancing Na^+^ toxicity. This complex network of ion transporters and pumps is critical for managing osmotic stress and maintaining cellular integrity under saline conditions. HKTs, K^+^ transporter; HAKs, K^+^ absorption transporter.

Plants alleviate sodium ion toxicity primarily by expelling sodium ions from cells and sequestering them via ion antiporters, such as NHX7 (also known as SOS1) in the plasma membrane and NHX1 in the vacuolar membrane.^116^ The activities of these ion antiporters are regulated by the calcium-dependent SOS2/SOS3 kinase complex.^117–119^ Na^+^ transport is driven by the proton motive force generated by H^+^-ATPase (located in the plasma and vacuolar membranes) and H^+^-VPPase (located in the vacuolar membrane).^120,121^ Salt stress enhances the activities of H^+^-ATPase and H^+^-VPPase.^122–124^ As a result, more H^+^ ions are pumped into the apoplast and vacuoles, increasing the transmembrane electrochemical gradient and enhancing the ability of Na^+^ to flow from the cytoplasm into the apoplast and vacuoles.^125,126^ Furthermore, the SOS2-SOS3 complex is sensitive to Ca^2+^ concentrations, and appropriate Ca^2+^ levels are beneficial for maintaining Na^+^ homeostasis under salt stress. ^104,127^ K^+^ is an essential nutrient in plant cells and is the most abundant cation,19 which is crucial for plant growth.^128,129^ Plants have different transport systems dedicated to acquiring K^+^, allowing them to promote K^+^ absorption across a wide range of external concentrations.^130^ Under salt-alkaline stress conditions, excessive Na^+^ influx into the cytoplasm can cause the membrane potential to drop below the resting potential.^131^ activating K^+^ efflux channels (e.g., NSCC), which disrupts the homeostasis of the K^+^/Na^+^ ratio. Increasing evidence suggests that several K^+^ channel proteins,^130,133^ including high-affinity K^+^ transporters (HKTs) and high-affinity K^+^ absorption transporters (HAKs), are involved in plant K^+^ uptake.^132^ HKT is a Na^+^/K^+^-specific transporter (Type II). ZmHKT2 encodes a transporter protein belonging to the K^+^-preferring HKT family.^133^ Its primary function is to reduce the K^+^ content in the shoot by actively removing K^+^ ions from the xylem sap flowing from the roots to the shoots.^134^ When *ZmHKT2* is absent, the concentration of K^+^ in the xylem sap and stem increases, ultimately leading to enhanced salt tolerance.^135^ In addition, the activity of H^+^-ATPase in the plasma membrane is another factor limiting K^+^ uptake. This protein complex is necessary for actively pumping protons out of the cell via an ATP-dependent phosphorylation process, generating a proton motive force (pmf) across the plasma membrane.^136^ The HAKs then use the pmf generated by H^+^-ATPase to absorb K^+^, as HAKs are typically K^+^/H^+^ symporters.^137–139^ Therefore, limiting membrane depolarization (by restricting Na^+^ influx or promoting Na^+^ efflux) and enhancing H^+^-ATPase activity can increase K^+^ absorption by HAKs under salt stress improving resistance to low K^+^ under such conditions.^140,141^ Thus, maintaining potassium ion homeostasis and sustaining a high K^+^/Na^+^ ratio are important adaptive strategies for salt stress (Figure 3).

Chloride (Cl^−^) is an essential micronutrient in plants.^142^ It regulates many physiological processes in plants, such as participating in photosynthesis, regulating the opening and closing of leaf stomata, and inhibiting the occurrence of diseases.^143,144^ In recent years, the role of Cl^−^ in plant salt tolerance has been reevaluated and has gained increasing attention.^145,146^ Compared to the in-depth studies of Na^+^ transport, research on Cl^−^ transport in maize under salt stress conditions is relatively limited. A recent study found that the A-type response regulator (ZmRR1) regulates Cl^−^ exclusion and salt tolerance in maize stems. Salt stress induces the degradation of *ZmRR1*, which relieves its inhibition of *ZmHP2*, thereby increasing the transcription of *ZmMATE29*, which promotes salt tolerance by compartmentalizing Cl^−^ in the vacuoles of root cortical cells, reducing the Cl^−^ load in the xylem of the stem and roots, and promoting Cl^−^ exclusion from the stem.^147,148^ The Cl^−^ concentration in the stems of salt-tolerant barley varieties is significantly lower than in sensitive varieties, indicating that controlling Cl^−^ accumulation in the stem is important for overall salt tolerance.^149^ Some studies have shown that wild-type soybeans have a stronger root Cl^−^ exclusion capacity than the relatively salt-sensitive cultivated soybean varieties, suggesting the important role of root Cl^−^ exclusion in soybean salt tolerance.^150,151^ In citrus rootstocks, salt-tolerant varieties with significantly lower Cl^−^ content in the stem mainly exhibit this tolerance due to stronger resistance of the roots to Cl^−^ absorption. Furthermore, studies have found a strong positive correlation between the transcript levels of *HvSLAH1* and *HvSLAC1* (anion channels that mediate Cl^−^ efflux) in barley leaves and grain yield.^152^

## 5. Conclusion and perspectives

The issue of soil salinization has become an increasingly serious concern due to a combination of factors, including the ongoing deterioration of the natural environment, the use of improper irrigation techniques, and the escalating impacts of climate change.^64,153,156^ These factors have resulted in the spread and intensification of salt accumulation in soils, which severely limits the ability of plants to grow and thrive. Salt stress is considered one of the most critical forms of abiotic stress, severely affecting plant development and productivity.^154,155^ It primarily restricts plant growth by disrupting key physiological processes, with the roots being the first organs to detect the presence of excessive salt in the soil.^156,157^ Once salt is detected, it triggers osmotic stress, as plants struggle to maintain water balance and proper cellular functions. In addition to osmotic stress, salt stress can lead to ion toxicity, which arises from the imbalance of essential nutrients and ions in the plant’s cytoplasm.^158^ This imbalance can inhibit various metabolic processes and compromise cellular function.^159,160^ To survive in such a harsh environment, plant cells must undergo a series of complex adaptive responses, including altering their internal structures and biochemical pathways to withstand the detrimental effects of salt stress.^161^

One of the most harmful consequences of salt stress is the generation of ROS, which are highly reactive molecules that can damage cellular components such as lipids, proteins, and DNA. The accumulation of ROS leads to oxidative stress, which further exacerbates cellular damage and disrupts ion homeostasis, ultimately impairing cellular metabolism and reducing plant vitality.^162,163^ In response to these damaging effects, plants activate their antioxidant defense systems to prevent the accumulation of ROS and minimize oxidative damage.^164,165^ This system is crucial for maintaining cellular integrity under stressful conditions. The activity of various antioxidant enzymes, including SOD, CAT, APX, and POD, increases as part of the plant’s strategy to mitigate oxidative stress.^166^

**Figure 4.**
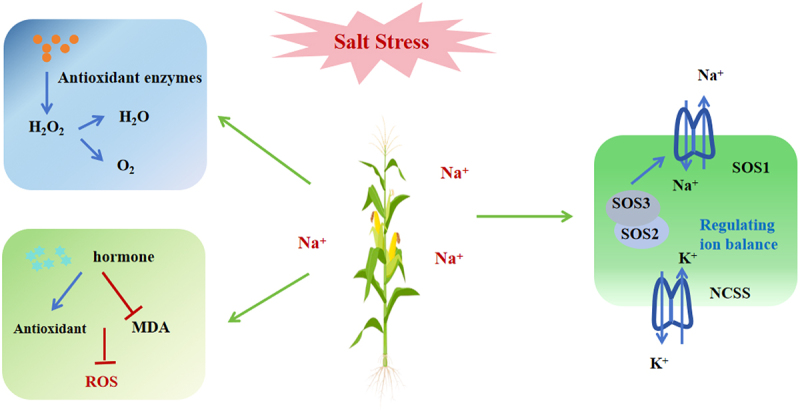
Under salt stress, maize employs a range of mechanisms to minimize cellular damage and maintain homeostasis. On one hand, maize produces excessive antioxidant compounds, which help neutralize ROS by converting hydrogen peroxide into water and oxygen, thus reducing oxidative damage. On the other hand, maize synthesizes an increased amount of hormones that suppress ROS production and regulate stress responses. Additionally, the SOS pathway is activated in maize to maintain ion balance, helping to manage the accumulation of toxic ions such as Na+ and ensuring cellular homeostasis under salt stress conditions.

Numerous studies have highlighted the critical role of ion transport and regulation of ion content in determining plant ability to salt stress tolerance.^167^ In saline environments, plants face the challenge of maintaining cellular homeostasis and proper physiological function despite the presence of excess salts in the soil. To achieve this, plants must carefully regulate ion fluxes across membranes, utilizing mechanisms such as selective absorption, active efflux, and ion compartmentalization within vacuoles and other cellular compartments. These strategies are vital for sustaining the dynamic balance required for normal metabolic processes, enabling plants to survive and thrive under salt stress conditions. One of the key players in salt tolerance is the HKT1 transporter, which has been shown to enhance salt tolerance by limiting the accumulation of sodium ions (Na^+^) in the stem tissues. By reducing Na^+^ buildup in the stem, HKT1 helps protect more sensitive tissues, such as the leaves, from the toxic effects of Na^+^. In addition to HKT1, plants also rely on ion antiporters, such as NHX7 (also known as SOS1) and NHX1, to expel excess sodium ions from the cytoplasm. These antiporters function in tandem with the proton motive force generated by the H^+^-ATPase and H^+^-VPPase pumps. The H^+^-ATPase and H^+^-VPPase enzymes create a proton gradient, which drives the activity of antiporters by using protons (H^+^) to actively transport Na^+^ out of the cytoplasm and into the vacuoles or extracellular spaces.^168,169^ The activity of SOS1 is regulated by the calcium-dependent SOS2/SOS3 kinase complex. To cope with salt stress, plants maintain a high sodium-to-potassium ratio. Under salt stress, the expression of genes encoding high-affinity K^+^ transport systems are upregulated, facilitating the expulsion of excess salts from the cells, thereby enhancing the plant’s salt tolerance (Figure 4).

Genomic editing technologies have revolutionized plant breeding by enabling precise and predictable modifications in plant genomes, allowing the development of crops with desired traits. Among these technologies, CRISPR/Cas9 stands out as one of the most advanced tools for genome engineering in crops.^170^ It has rapidly expanded in application, being utilized in major crops such as rice, wheat, and maize, as well as other essential food security crops like potato and cassava. The precision and efficiency of CRISPR/Cas9 allow for targeted modifications that can enhance crop traits, including stress tolerance, yield, and nutritional content.

For example, CRISPR/Cas9 has been used to create *ZmSWEET1b* knockout maize lines, resulting in significant reductions in fructose and sucrose content. This modification also led to a decrease in the expression of genes involved in sodium (Na^+^) efflux, including *ZmSOS1*, *ZmH*^*+*^*-ATPase 2*, and *ZmH*^*+*^*-ATPase 8*, which contributed to a salt-sensitive phenotype.^171^ Similarly, a study on maize identified an ethylene response factor, ZmEREB57, which, when knocked out using CRISPR/Cas9, reduced salt tolerance. This reduction was linked to inhibited synthesis of 12-oxo-phytodienoic acid (OPDA) and jasmonic acid (JA), both of which are involved in stress responses.^76^ In another study, the CRISPR/Cas9-mediated knockout of the salt-responsive gene *ZmCLCg* in maize resulted in a salt-sensitive phenotype characterized by significant reductions in root and stem growth under NaCl treatment. These examples demonstrate the potential of CRISPR/Cas9 in identifying and manipulating genes crucial for salt tolerance in maize.^172^

In addition to genomic editing, traditional genetic approaches, such as quantitative trait locus (QTL) mapping, remain integral to understanding the genetic basis of traits like salt tolerance.^173^ QTL mapping involves grouping a population of plants based on molecular markers and comparing phenotypic differences between genotypes. This technique helps locate target genes by associating specific markers with desirable traits.^174^ While much QTL research has focused on rice and wheat, a few salt tolerance QTLs have been identified in maize. For instance, a major QTL for mature plant height was detected on chromosome 1, where two candidate genes related to ion homeostasis were found. Additionally, a salt tolerance QTL was associated with the ZmHKT1 gene, which encodes a transporter responsible for excluding sodium (Na^+^) from leaves by extracting it from the xylem sap, thereby contributing to salt tolerance.^175^

Genome-wide association studies (GWAS) have become increasingly valuable for identifying genes that control complex traits by examining the linkage disequilibrium (LD) between molecular markers and candidate genes across the genome.^176^ In maize, a GWAS study involving 445 natural accessions under salt stress identified 49 candidate genes at 57 loci associated with salt tolerance. Approximately 44% of these genes were related to ABA signaling, auxin signaling, stomatal closure, and other stress responses, highlighting their role in maize’s response to salt stress.^177^ Another GWAS study using 305 maize inbred lines under salt stress identified seven significant single nucleotide polymorphisms (SNPs), which led to the identification of 120 genes, including two that regulate ion transport. These findings further confirm the importance of ion transporters in enhancing maize salt tolerance, underscoring the value of GWAS in pinpointing genes that contribute to stress resilience.^178^

Together, the combination of CRISPR/Cas9 technology, QTL mapping, and GWAS is advancing our understanding of the genetic mechanisms underlying salt tolerance in maize. These approaches provide valuable insights into the identification and manipulation of genes that can be used to breed salt-tolerant crops, which is essential for improving food security in regions affected by salinity stress.

Although significant progress has been made in understanding salt tolerance in maize, including the identification and validation of key genes and signaling pathways involved in salt stress responses ((Supplementary table 1 and Supplementary table 2), there remains much to be uncovered. The complex networks of gene regulation, protein interactions, and metabolic pathways that orchestrate maize’s response to salinity are still not fully understood. Without a clear understanding of the underlying genetic and molecular mechanisms, breeding efforts are hindered, making it difficult to identify the most effective targets for selection. Traditional breeding methods alone may not be sufficient to overcome the challenges posed by salinity, especially in the context of rapidly changing environmental conditions due to climate change. To successfully develop salt-tolerant maize varieties, researchers must explore the genetic diversity present within maize populations, as well as the potential for adaptive traits in different ecological environments. This requires not only identifying candidate genes that confer salt tolerance but also understanding how these genes are expressed and regulated under salt stress conditions.

## Supplementary Material

Supplementary table. 2.docx

Supplementary table. 1.docx
