# Supplementary material for: Advances in deciphering the mechanisms of salt tolerance in Maize
Source: Plant Signal Behav. 2025 Mar 18;20(1):2479513. doi: 10.1080/15592324.2025.2479513 (PMC11959903; doi:10.1080/15592324.2025.2479513)
Supplement: Supplementary table. 2.docx [file KPSB_A_2479513_SM1870.docx]

**Supplementary table. 2 Varieties and genotypes important for investigation in respect to salt tolerance**

| Salt-tolerant corn varieties | Salt-tolerant maize inbred line |
| --- | --- |
| YD9 | PH6WC |
| WC937 | Ye3189 |
| GD0810 | Chengxi53 |
| GD920 | Chang7-2 |
| GD908 | X178 |
| XD903 | Qi319 |
| RF319 | Zheng58 |
| QQ515 | Jing724 |
| TPY891 | Ye478 |
| YDY11 | K10 |
| WC1306 | Mo17 |
| QQ921 | Jing92 |
| LH200 | PH4CV |
| KN20 | B73 |
| LR888 | PHPR5 |
| LR999 |  |
| JF399 |  |
| LH151 |  |
| TG88 |  |
| GD3121 |  |
