# Supplementary material for: Advances in deciphering the mechanisms of salt tolerance in Maize
Source: Plant Signal Behav. 2025 Mar 18;20(1):2479513. doi: 10.1080/15592324.2025.2479513 (PMC11959903; doi:10.1080/15592324.2025.2479513)
Supplement: Supplementary table. 1.docx [file KPSB_A_2479513_SM1869.docx]

**Supplementary table. 1 Some major genes, transcription factors pertaining to salt tolerance**

| **Transcription factors involved in Salt-tolerant** | **Proteins involved in Salt-tolerant** |
| --- | --- |
| NAC transcription factors（ZmNAC55、ZmNAC11） | Na^+^/H^+^ antiporters（NHX）: ZmNHX1 |
| bZIP transcription factors（ZmbZIP72） | K^+^ transporter(HKT): ZmHKT1 |
| MYB transcription factors（ZmMYB48） | Late embryonic protein (LEA): ZmLEA3 |
| WRKY transcription factors（ZmWRKY58） | [heat shock protein](javascript:;)(HSP): ZmHSP70 |
| DREB/CBF transcription factors（ZmDREB2A） | Osmoregulatory substance synthesis related proteins ([proline](javascript:;)、[betaine](javascript:;)): ZmP5CS1、ZmBADH1 |
| HD-Zip transcription factors（ZmHB53） | [antioxidase](javascript:;)：SOD、POD、CAT、APX |
| bHLH transcription factors（ZmPTF1） | K^+^ [channel protein](javascript:;): ZmAKT1 |
| MADS-box transcription factors（ZMSOC1） | [calmodulin](javascript:;)（CaM）: ZmCaM1、ZmCaM22 |
| YABBY transcription factors（ZmYAB1） | MAPK cascade reactive protein: ZmMAPK1、ZmMAPK2 |
| Zinc finger transcription factors（ZmC2H2-1） | [aquaporins](javascript:;)（AQP）: ZmPIP1 |
| WOX transcription factors（ZmWOX5） | Proteases and protease inhibitors: ZmCysP1、ZmPI1 |
